# Supplementary figures and images for: Influence of sex, season and environmental air quality on experimental human pneumococcal carriage acquisition: a retrospective cohort analysis
Source: ERJ Open Res. 2022 Apr 11;8(2):00586-2021. doi: 10.1183/23120541.00586-2021 (PMC8995542; doi:10.1183/23120541.00586-2021)

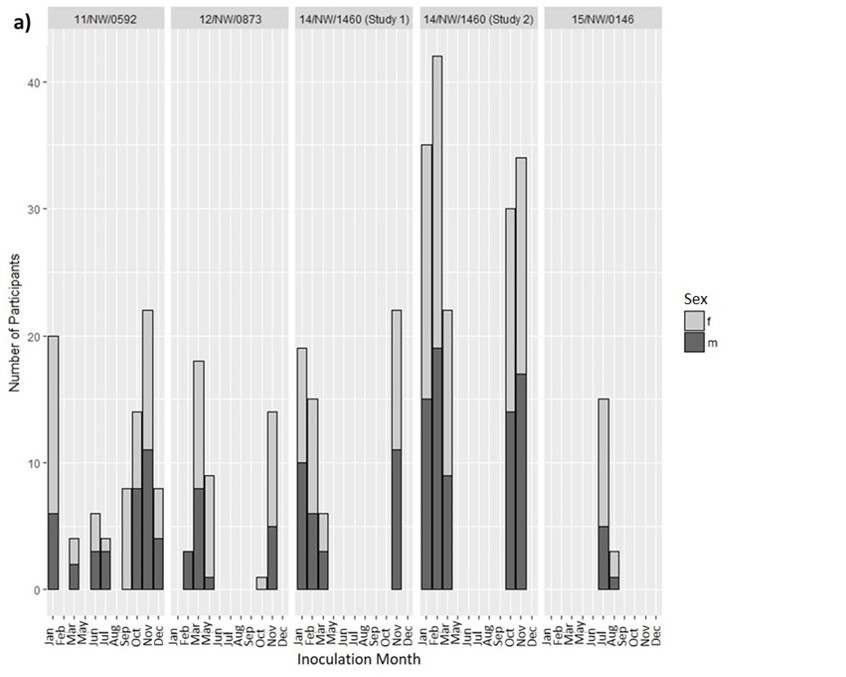

Supplement: Supplementary file 1 [file 00586-2021.FIGURES1.jpg]

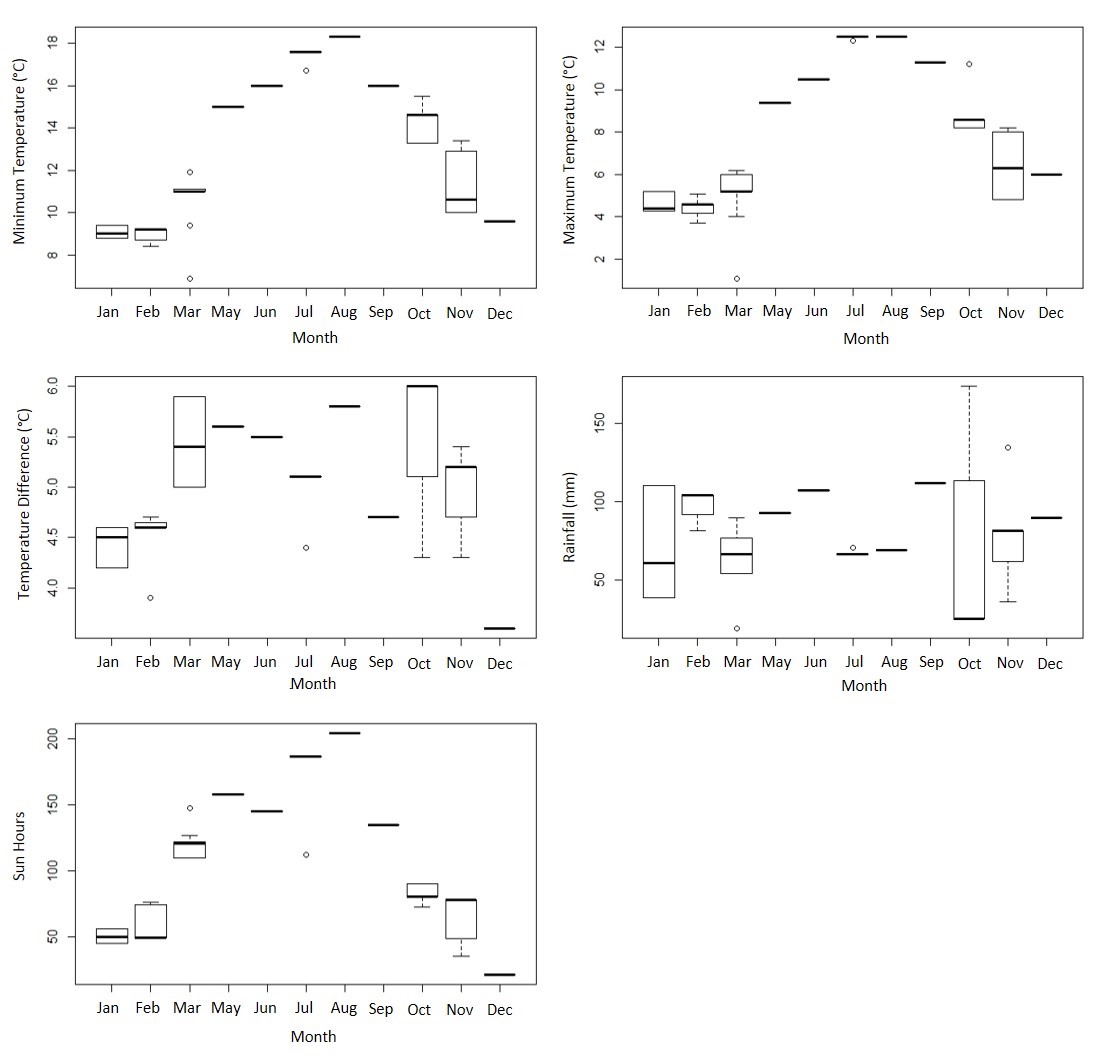

Supplement: Supplementary file 2 [file 00586-2021.FIGURES2.jpg]

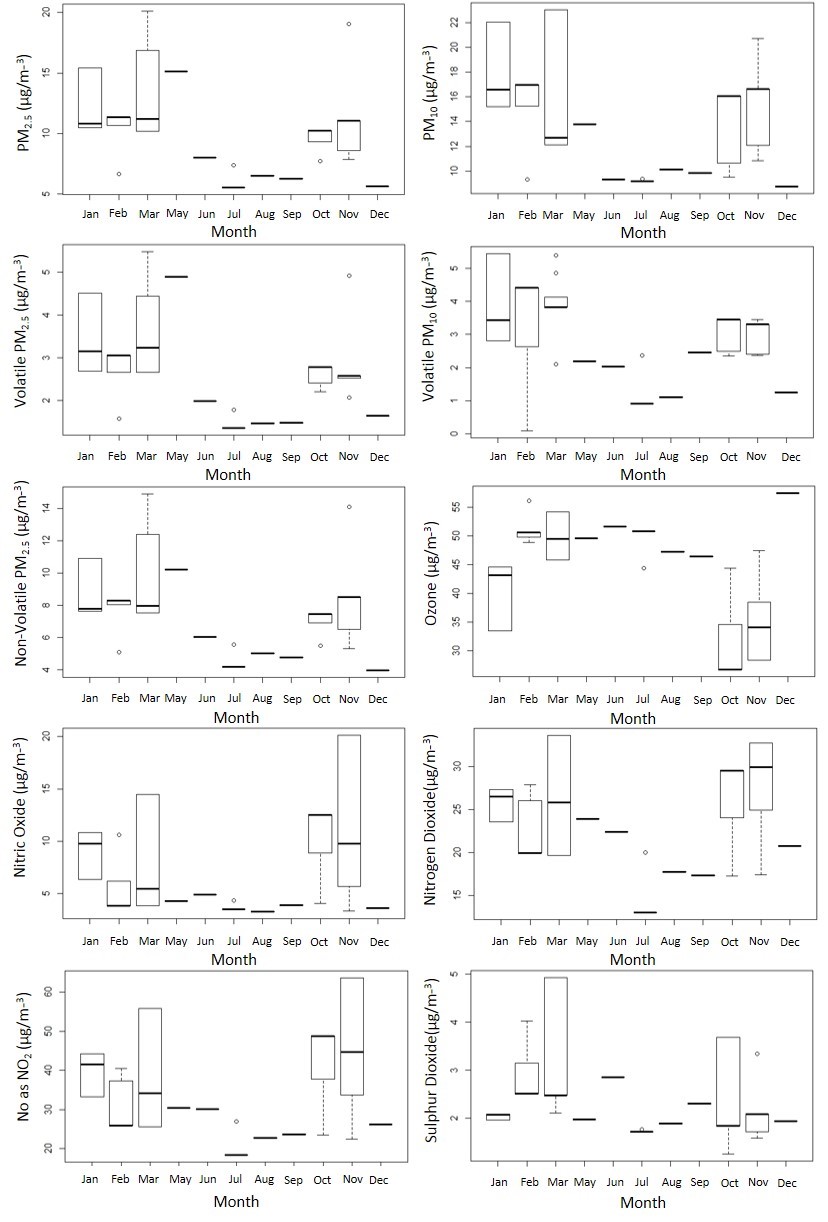

Supplement: Supplementary file 3 [file 00586-2021.FIGURES3.jpg]

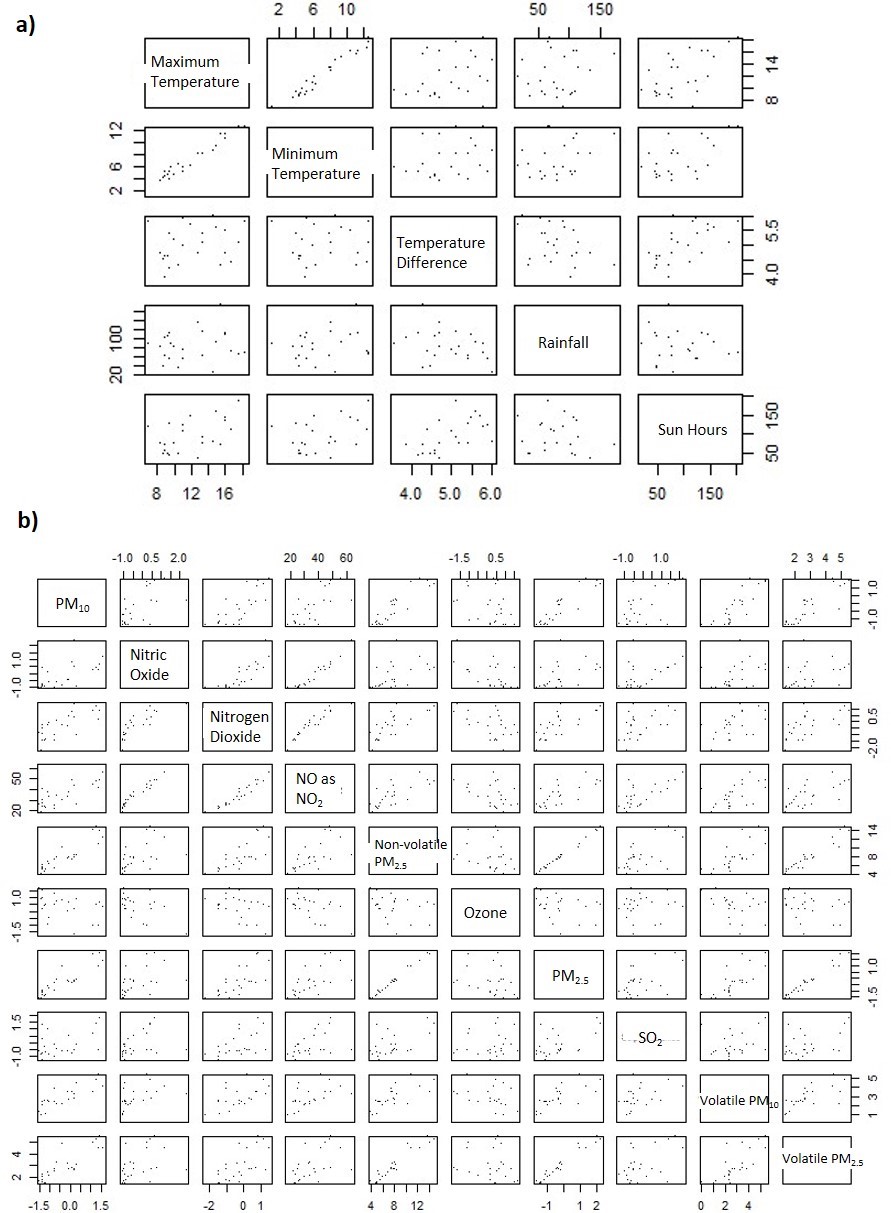

Supplement: Supplementary file 4 [file 00586-2021.FIGURES4.jpg]

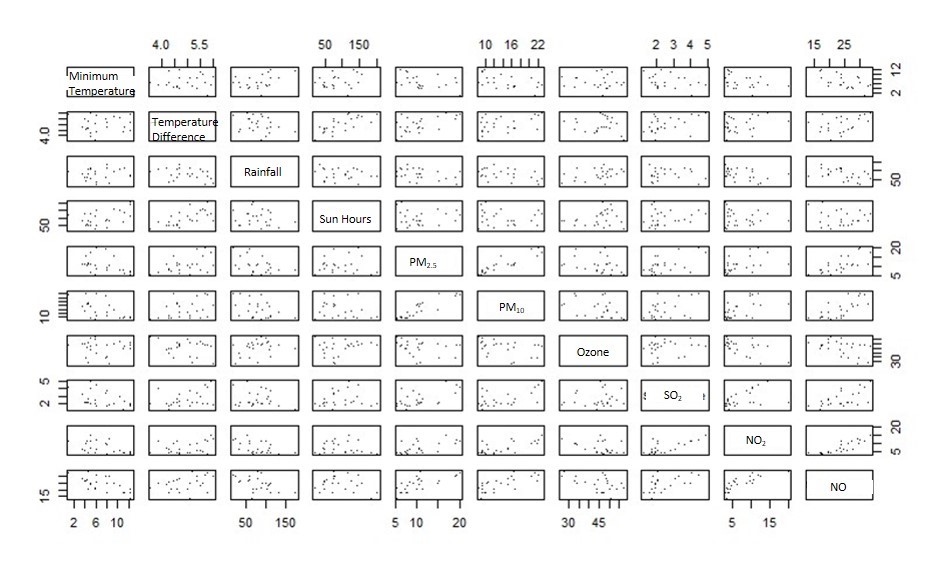

Supplement: Supplementary file 5 [file 00586-2021.FIGURES5.jpg]
